# Evaluation of bacteriophage efficacy against *Pseudomonas aeruginosa* in ex vivo and in vitro canine skin systems

**DOI:** 10.1038/s41598-026-40091-8

**Published:** 2026-02-17

**Authors:** Anne Dalponte, Viviane Filor, Andreas Nerlich, Mathias Müsken, Marcus Fulde, Wolfgang Bäumer

**Affiliations:** 1 Institute of Pharmacology and Toxicology, School of Veterinary Medicine, Freie Universität Berlin, Berlin, Germany; 2 Veterinary Centre for Resistance Research (TZR), School of Veterinary Medicine, Freie Universität Berlin, Berlin, Germany; 3 Central Facility for Microscopy (ZEIM), Helmholtz Centre for Infection Research, Braunschweig, Germany; 4 Institute of Microbiology and Epizootics, School of Veterinary Medicine, Freie Universität Berlin, Berlin, Germany; 5 Present Address: Institute of Microbiology, University of Veterinary Medicine Hannover, Foundation, Hannover, Germany

**Keywords:** Pseudomonas aeruginosa, Bacteriophage therapy, Epidermal equivalent, Franz-type diffusion cell, Biological techniques, Biotechnology, Diseases, Microbiology

## Abstract

*Pseudomonas aeruginosa* is an opportunistic pathogen responsible for chronic infections in both human and veterinary medicine, with biofilm formation and multidrug resistance posing major clinical challenges. The efficacy of two bacteriophages, JG003 and PTLAW1, alone and in combination, was evaluated. In vitro biofilms grown on abiotic 96-well plates showed significant reduction after treatment with individual bacteriophages or their combination, as confirmed by confocal microscopy. To better simulate physiological conditions, efficacy was assessed using an epidermal equivalent model and an ex vivo canine skin model. In the ex vivo system, bacteriophage treatment reduced bacterial load by 4 logs, as confirmed by scanning electron microscopy and immunofluorescence imaging. In the epidermal equivalent model, bacteriophage therapy decreased bacterial counts and CXCL8 levels without inducing cytotoxicity or disrupting the skin barrier. Integration of in vitro and ex vivo systems bridges the gap between traditional biofilm assays and in vivo studies. The use of Franz-type diffusion cells provides a physiologically relevant platform for evaluating topical bacteriophage delivery and skin permeation. These findings establish a reproducible preclinical framework for biofilm-targeted therapies, demonstrating that bacteriophage combinations effectively reduce *Pseudomonas aeruginosa* biofilms and inflammation on skin, supporting their potential for wound treatment in both human and veterinary medicine.

**Supplementary Information:**

The online version contains supplementary material available at 10.1038/s41598-026-40091-8.

## Introduction

*Pseudomonas aeruginosa* (*P. aeruginosa*) is an opportunistic pathogen that presents significant challenges in both human and veterinary medicine. The infections are difficult to treat due to their intrinsic, acquired and adaptive resistances to antibiotics^1,2^. *P. aeruginosa* predominantly exists as biofilm, which contributes to antibiotic tolerance, shielding bacteria from immune clearance and drug exposure through physical barriers, dormancy and stress response activation^3^. These factors contribute to disease progression and are associated with the establishment of chronic infections^4^. Due to its global prevalence, resistance potential, and clinical impact, multidrug-resistant *P. aeruginosa* is listed as a high-priority pathogen on the World Health Organization’s 2024 Bacterial Priority List^5^. In human medicine *P. aeruginosa* is a major cause of healthcare-associated, life-threatening infections such as pneumonia, septicemia, and post-surgical complications. Infections in critically ill or immunocompromised individuals are especially severe and often lead to significant morbidity^1^.

Within the framework of the One Health approach, which emphasizes the interdependence of human, animal, and environmental health, *P. aeruginosa* is increasingly recognized as a zoonotic threat capable of spreading across species and ecological boundaries. A recent One Health study from northern Portugal analysed 737 *P. aeruginosa* isolates from human, animal, and aquatic sources. The researchers identified shared high-risk clones across these reservoirs, providing clear evidence of transmission^6^.

The accelerating rise in antimicrobial resistances is due to the overuse and misuse of antibiotics in both human and veterinary medicine. Given the limitations of conventional antibiotics in treating biofilm-associated infections, especially those involving multidrug-resistant strains, bacteriophages (phages) have emerged as a promising therapeutic option^7^. Phage therapy has been applied in both humans and animals since the early 20th century. While its use declined in Western countries with the rise of antibiotics, it remained common in Eastern Europe, notably in Georgia^8^. In recent years, the increasing prevalence of multidrug-resistant bacterial infections has renewed worldwide interest in phage therapy. Due to their high host specificity and natural abundance, it is often possible to isolate effective phages from environmental sources that can lyse even highly resistant strains. Moreover, several studies have reported that phage treatment can resensitise bacteria to antibiotics, enhancing the overall therapeutic effect^7^.

Phages could also be a game-changer in treating biofilms: Many phages produce biofilm-degrading enzymes, such as depolymerases, which break down the extracellular matrix and enhance phage and antibiotic penetration^9^. These enzymes, often located on tail fibers or spikes, help phages access bacterial cells by degrading capsular and exopolysaccharides. Some depolymerases are effective even when applied alone, disrupting mature biofilms and dispersing bacterial cells^10,11^. In addition, phages can navigate through biofilm structures via natural channels, allowing them to reach deeper bacterial populations^12^. These adaptations highlight the potential of phage therapy to overcome the limitations of traditional antibiotics in treating multidrug-resistant *P. aeruginosa.* However, the models on abiotic surfaces like well plates, often lack the complexity of host tissue.

Franz-type diffusion cells, used in this study with canine skin, more closely mimic the infection mechanism on the skin’s surface. The surface of the skin consists of a cornified layer of proteins and unique lipids, dotted with glands and hair follicles^13,14^, which play an important role for a pathogen infection in the skin and the expression of virulence factors in bacteria. For instance, *P. aeruginosa* exhibits different gene expression patterns in clinically relevant environments, such as burn wound exudate or the cystic fibrosis lung^15,16^. Similarly, *P. aeruginosa* undergoes genetic adaptation in the cystic fibrosis lung, including mutations in regulatory genes such as *lasR*, promoting biofilm formation and antibiotic resistance^17^.

These host-specific adaptations emphasise the need for experimental models that better replicate physiological conditions. While in vivo models offer valuable insights into infection dynamics and host responses, they are limited by ethical concerns, high costs, species-specific differences, and low throughput, making them less suitable for early-phase screening of antimicrobial agents^18^.

The Franz-type diffusion cells are widely employed to simulate the penetration of compounds through biological membranes such as skin and their efficacy^19^and consist of donor and receptor chambers separated by a membrane like excised skin, allowing for controlled application of antimicrobial agents on the skin surface. The advantage of using Franz-type diffusion cells compared to conventional well plates in ex vivo experiments lies in the guaranteed apical substance application and have the ability to evaluate skin permeability for various compounds, including phages. Ex vivo models have been widely used for infection studies^20,21^, with some investigations focusing on the efficacy of phage therapy under physiologically relevant conditions, revealing a decrease in bacterial burden^22–24^.

For phages, however, pharmacokinetic data are limited, and studies have shown that intact skin represents a formidable barrier, resulting in poor phage penetration and bioavailability^25^. This highlights that intact skin serves as a barrier against phage permeation, which could restrict the effectiveness of topical phage therapy unless the skin is compromised or specific formulation strategies are employed. Therefore, infection models that closely mimic physiological or damaged skin environments are critical to understanding phage penetration and antimicrobial efficacy in clinically relevant settings.

To address the limitations of conventional biofilm models and the clinical need for alternatives to antibiotics, we designed this study to evaluate whether a defined phage combination (JG003 + PTLAW1) can (i) disrupt pre-formed *P. aeruginosa* biofilms, (ii) reduce bacterial burden in skin tissues, and (iii) modulate inflammation in the absence of cytotoxicity. By applying both a Franz-type ex vivo skin model and an in vitro epidermal equivalent, we tested the phage efficacy in a biologically relevant context and assessed their translational potential in treating skin infections.

## Results

### Phage treatment significantly reduced biofilm volume and bacterial viability while increasing membrane-compromised cells in confocal analysis

To assess the effect of the phage treatment (JG003, PTLAW1 and combination) on pre-formed biofilms, BacLight Live/Dead staining was performed, and biofilm volumes were quantified using confocal microscopy and 3D image analysis.

**Fig. 1:**
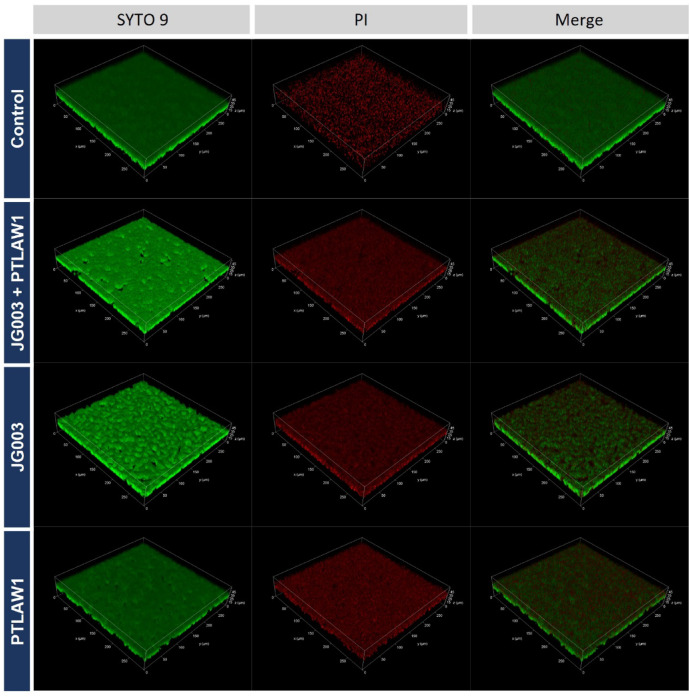
Representative 3D confocal microscopy images of pre-formed *P. aeruginosa* biofilms treated with phages. Biofilms were grown for 16 h, following the treatment with JG003, PTLAW1 and their combination at a final concentration of 1 × 10^9^ PFU ml^−1^. LB-media was used as a control. BacLight LIVE/DEAD staining (SYTO 9 = green, viable cells; PI = red, membrane-compromised cells) was used to assess biofilm viability and structure. One representative out of three independent experiments is shown. Phage-treated groups display visible reduction in biofilm density and increased PI signal, indicating membrane damage.

**Fig. 2:**
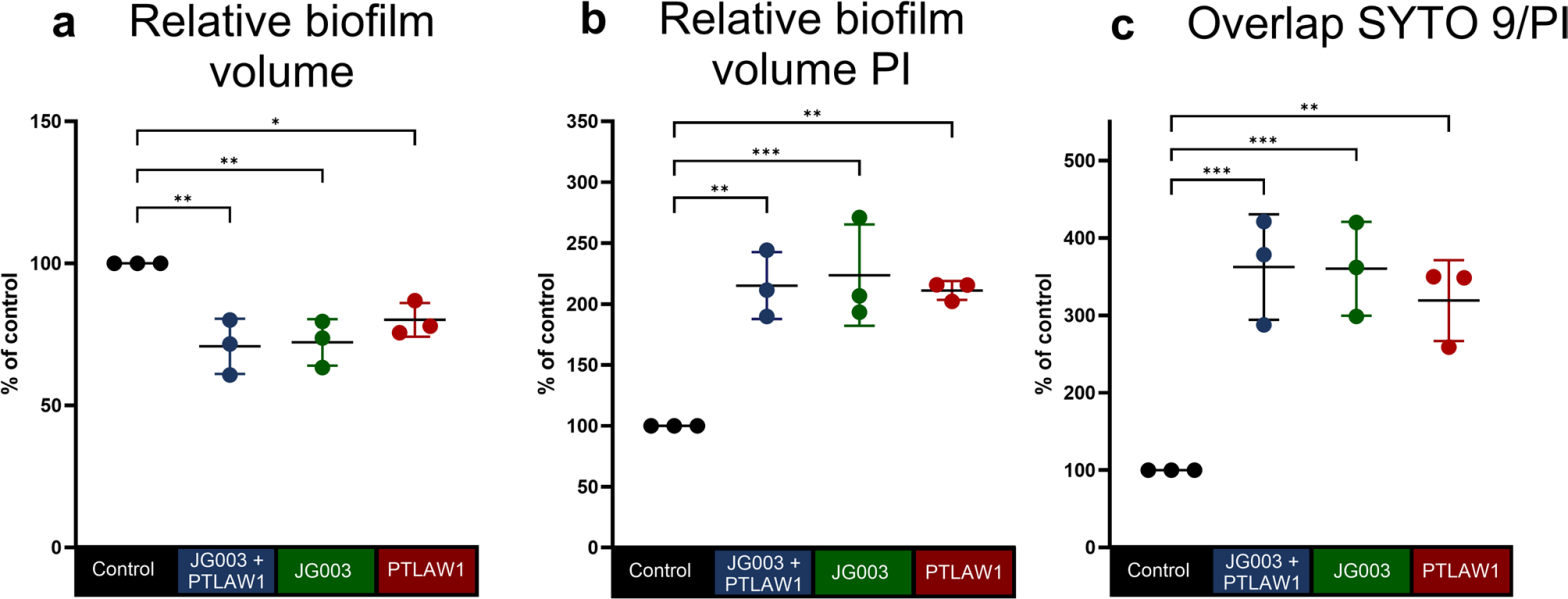
Quantitative confocal analysis of *P. aeruginosa* biofilms after phage treatment. Biofilms were grown for 16 h and treated with phage JG003, PTLAW1 and the combination (JG003 + PTLAW1) at a final concentration of 1 × 10^9^ PFU ml^−1^. LB-media was used as a control. Quantification of the confocal scanning microscope images of *P. aeruginosa* biofilms with BacLight LIVE/DEAD staining. (**a**) Relative biofilm volume (**b**) Relative, membrane-compromised biofilm volume (PI). (**c**) Relative, overlapping biofilm volume (co-occurrence of SYTO 9 and PI objects within the relative biofilm volume). Control values were set to 100%, and treated groups are expressed as percentage of control. Each dot represents a biological replicate (*n* = 3). Statistical analysis was performed using ordinary one-way ANOVA with Dunnett’s multiple comparison test; *p < 0.05, **p < 0.01, ***p < 0.001.

As shown in Fig. 1, phage-treated groups display visible reduction in biofilm density and increased PI signal, indicating membrane damage. Quantification of the biofilm volumes revealed a significantly reduced volume in all the phage-treated groups compared to the control. In particular treatment with JG003 resulted in a decrease by 27.79% to 72.21% (*p* = 0.0033), while the combination treatment reduced the volume by 29.16% to 70.84% (*p* = 0.0024) compared to the control volume (Fig. 2A). Treatment with PTLAW1 alone also reduces the biofilm volume by 19.87% to 80.13% (*p* = 0.0211) compared to the control.

In contrast, the volume of the PI-stained biofilm (Fig. 2B), indicating membrane-compromised cells, was significantly elevated in all phage-treated groups. JG003 and the combination treatment increased PI-stained volume by 123.7% and 115.2%, respectively (both *p* < 0.002). The PTLAW1 treatment resulted in the increase, with a mean difference of 111.2% (*p* = 0.0017) compared to control, suggesting substantial cell damage.

The overlapping volume (Fig. 2C) represents the portion of biofilm volume positive for both SYTO 9 and PI signal. This metric is based on segmented volumetric objects (relative to the total biofilm volume) and was low in the control group, indicating minimal overlap between live (green) and membrane-compromised (red) bacteria. The overlapping proportions were increased in the JG003-treated group by 260.2% to 360.2% (*p* = 0.0008), and were significantly increased in the combination-treated group (262.3%, *p* = 0.0007) compared to the control. The PTLAW1 treatment resulted in the increase, with 219.0% (*p* = 0.0024) compared to the control, reflecting a higher proportion of membrane-compromised cells.

### Phage combination treatment reduces bacterial load and disrupts biofilm structure on skin in an ex vivo experiment using the Franz-type diffusion cells

**Fig. 3:**
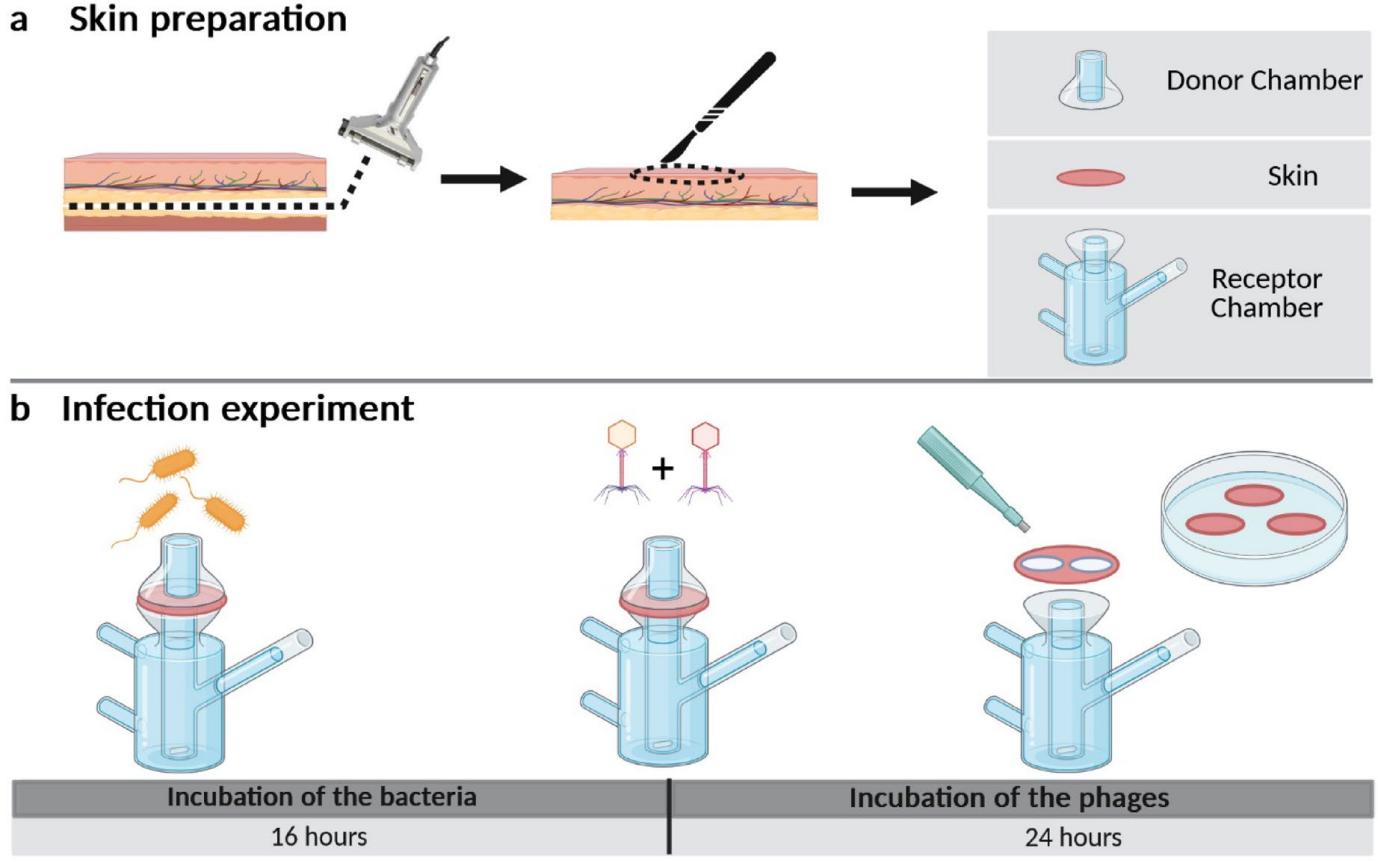
Ex vivo infection experiment workflow with the Franz-type diffusion cells. (**a**) Skin preparation: skin ablation with an electronic dermatome, sectioned to an appropriate diameter, clamped between the donor and receptor chamber. (**b**) Infection experiment: skins were infected with *P. aeruginosa* and incubated for 16 h. Treatment with phages and incubation for additional 8 h. Sampling with a 6 mm biopsy punch.

After processing canine skin biopsies, the top layers (set to 700 μm thickness) were clamped in Franz-type diffusion cells, infected with 4 × 10^7^ CFU *P. aeruginosa* for 16 h, and treated with PBS or the phage cocktail (JG003 + PTLAW1) for additional 8 h (Fig. 3). Throughout the experiment, the temperature of the skin surface was constant at 31.4 ± 0.76 °C. The weight of all 6 mm skin biopsies samples was consistent (34.7 ± 10.2 mg), with a thickness of 870.67 ± 110.98 μm.

**Fig. 4:**
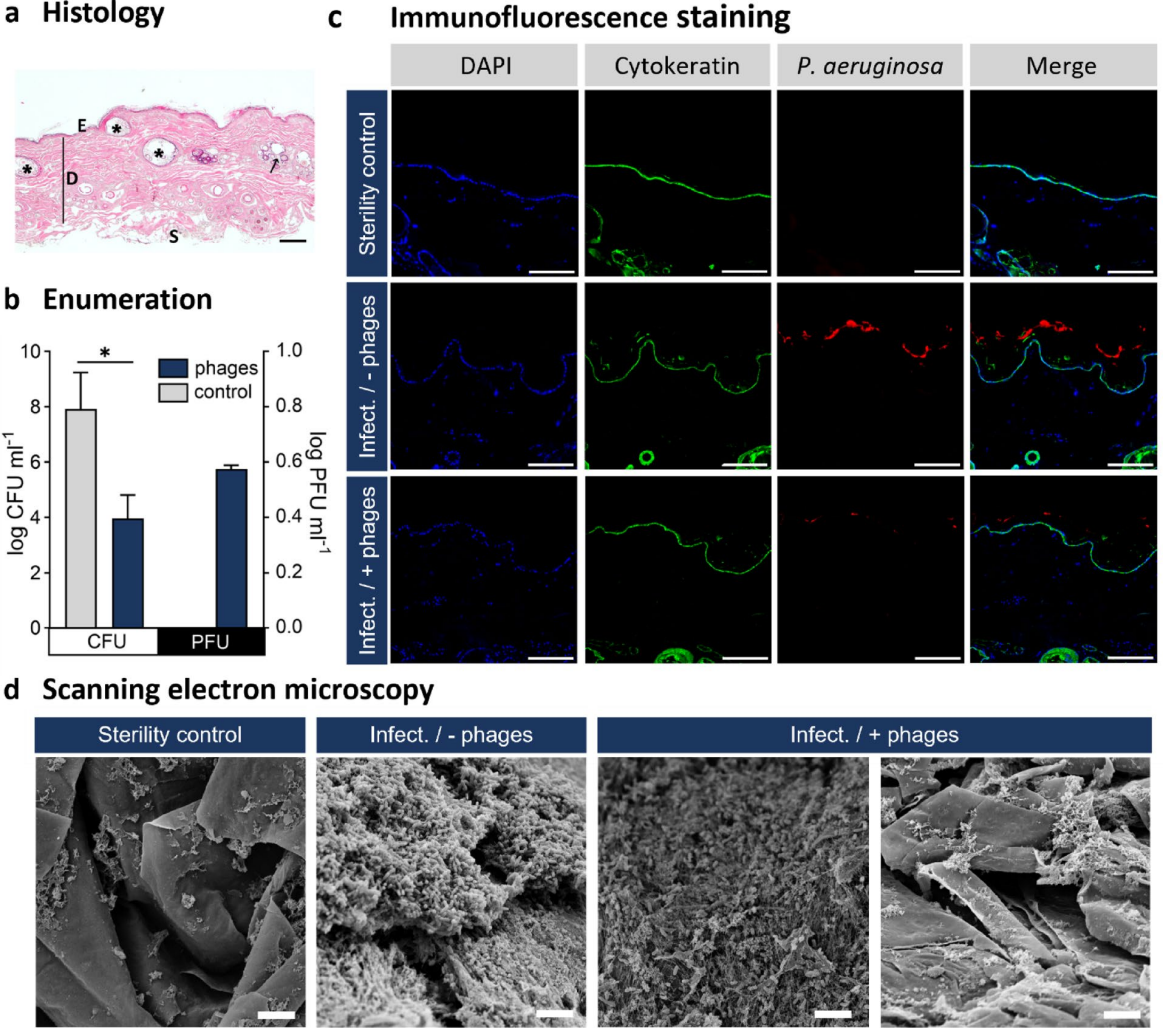
Evaluation of phage treatment in the ex vivo canine skin model using Franz-type diffusion cells. The skin samples were clamped into the Franz-type diffusion cells and infected with *P. aeruginosa* for 16 h, followed by treatment with the phage combination JG003 + PTLAW1 for 8 h. (**a**) Representative H&E staining of the control skin sample. E: Epidermis; D: Dermis; S: Subcutis; * Follicle. (**b**) Quantification of the bacterial (CFU ml^−1^) and phage levels (PFU ml^−1^) in homogenised skin samples. Experiments were performed in 3 biological replicates. (**c**) Representative immunofluorescence staining of skin cross-sections using anti-*P. aeruginosa* antibodies (red), anti-Pan-cytokeratin antibodies (green), and DAPI (blue). (**d**) SEM micrographs showing skin surface biofilm morphology in control and treated samples. Scale bars: (**a**) 200 μm; (**c**) 50 μm; (**d**) 5 μm.

The haematoxylin and eosin (H&E) staining (Fig. 4A) of excised canine skin revealed the typical architecture of physiological skin, with a stratified and cornified epidermis, a dermis with connective tissue, blood vessels, and adnexal structures such as hair follicles, as well as a subcutis composed of adipose tissue.

The phage combination revealed a significant drop (*p* < 0.05) in the bacterial counts by 4 log levels compared to the infected, not treated group (Fig. 4B). In all homogenised infected phage-treated skin samples, phages were detected at a concentration of 5.75 ± 0.11 log PFU ml^−1^, showing that the phages remained active over the duration of the experiment. No phages were detected in the acceptor buffer. These findings were supported by imaging:

Immunofluorescence staining was used to visualise both the epidermis and *P. aeruginosa*. The anti-Pan cytokeratin antibody stains most acidic (type I) and basic (type II) cytokeratin of the epidermis, defining the border of the skin layers. DAPI visualizes the cell nuclei, mainly in the stratum basale, while anti-Pseudomonas antibodies enabled the detection of the bacteria. We observed a stronger fluorescent signal of the *P. aeruginosa* antibody in the infected, not treated group compared to the infected phage-treated group. The fluorescence staining revealed bacterial presence on the surface of the stratum corneum as well as within deeper epidermal layers (Fig. 4C). The bacteria were predominantly organized in aggregated colonies, evidenced by areas of intense fluorescence signal, consistent with bacterial aggregation. SEM images (Fig. 4D) showed similar bacterial aggregates and extracellular matrix on the skin surface. In the sterility control, no bacterial structures were observed and the skin surface appeared physiological, showing the characteristic skin scales and hair. In contrast, the infected, not treated group exhibits bacterial clusters adhering to the skin surface, embedded in an amorphous extracellular matrix. The infected phage treated sample showed both, regions where the biofilm structure was absent, interspersed with areas with bacterial clusters and matrix components. Additional SEM images at different magnifications are provided in Supplementary Fig. 1.

### Phage treatment limits early *P. aeruginosa* biofilm formation and the inflammatory response in a canine epidermal equivalent model

**Fig. 5:**
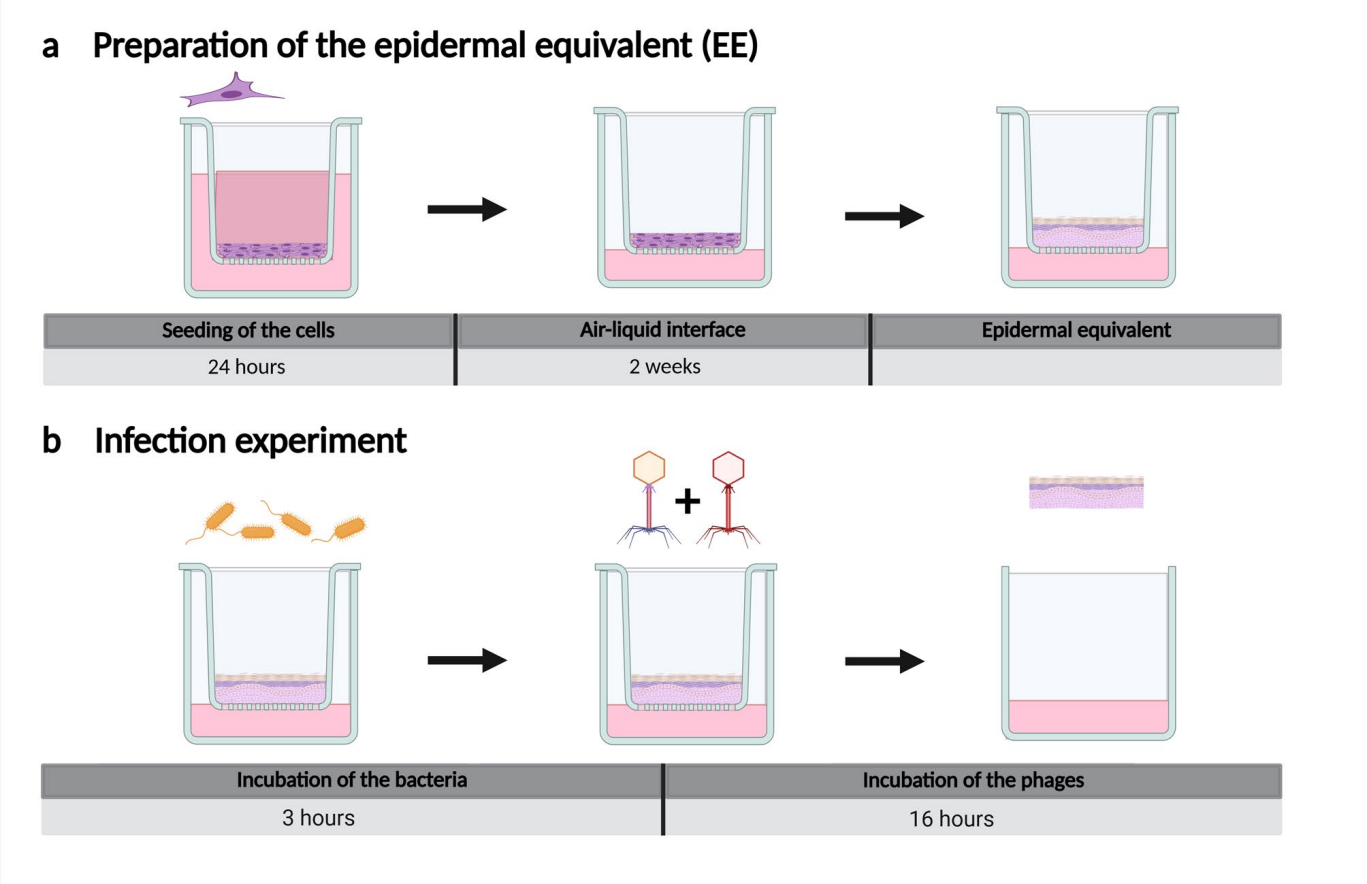
In-vitro infection experiment workflow of the canine epidermal equivalent. (**a**) Canine Epidermal Keratinocyte Progenitors (CPEK) were seeded in a transwell insert system and incubated at an air liquid interface (ALI) for 2 weeks. (**b**) Infection experiment: EE were infected with *P. aeruginosa* and incubated for a total of 16 h. The phage combination treatment was applied after 3 h.

Canine keratinocytes were cultured at an air-liquid interface in a transwell insert for 14 days, before starting the infection experiment with 1 × 10^3^ CFU/transwell. After 3 hours, the transwells were treated with 30 µl of 1 × 10^9^ PFU ml^−1^ of the phage combination JG003 + PTLAW1 and further incubation for a total of 16 h (Fig. 5). Preliminary experiments testing the cytotoxicity and viability of the phages on CPEK monolayers revealed no cytotoxic effects. The experimental setup and results are provided in Supplementary Text S1 and Supplementary Fig. 2.

**Fig. 6:**
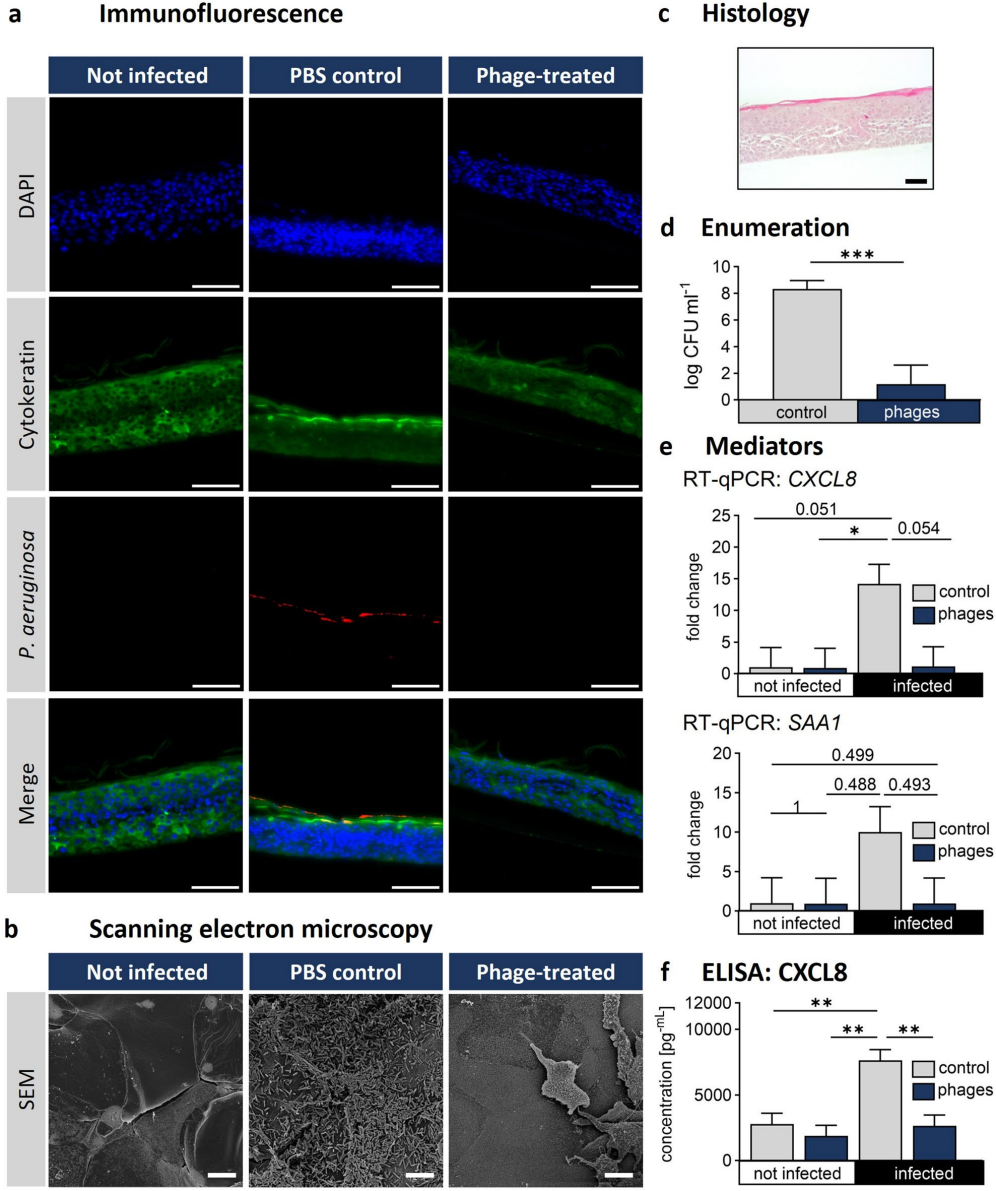
Evaluation of phage efficacy towards *P. aeruginosa* in the canine epidermal equivalent (EE) model. Canine keratinocytes were cultured in a transwell ALI system for 14 days and infected with *P. aeruginosa* (1 × 10^3^CFU). After 3 hours, the phage cocktail (JG003 + PTLAW1, 1 × 10^9^ PFU ml^−1^) was applied and incubated for an additional 13 h. (**a**) Representative immunofluorescence staining. (**b**) Scanning electron microscopy of the canine skin sample. (**c**) Representative H&E staining of the control epidermal skin equivalent. (**d**) Quantification of *P. aeruginosa* (log CFU ml^−1^), *n* = 3. (**e**) Quantitative reverse transcriptase PCR. Statistical analysis was performed using a linear mixed-effects model with Bonferroni correction. *p < 0.05. *n* = 3; (**f**) CXCL8 concentration by ELISA. *p < 0.05. *n* = 3. Scale bars: (**c**) 200 μm. (**e**) 50 μm. (**f**) 10 μm.

Immunofluorescence staining was used to visualize the epidermal equivalent and *P. aeruginosa*. Pan-cytokeratin staining defined the epidermal layers, while DAPI labeled cell nuclei and anti-*Pseudomonas* antibodies enabled bacterial detection. A stronger fluorescent signal for *P. aeruginosa* was observed in the infected, PBS control group compared to the infected, phage-treated group (Fig. 6A).

The SEM analysis revealed a dense bacterial biofilm in the PBS control group, with bacteria embedded in a matrix. The phage-treated group displayed the equivalent surface with areas with scattered bacteria (Fig. 6B). Additional SEM images at different magnifications are provided in Supplementary Fig. 3. The histological analysis of the EE confirmed the stratified structure of the canine epidermis, with defined epidermal layers and a stratum corneum without nuclei (Fig. 6C). The enumeration of the epidermal equivalent showed a mean log_10_ 8.33 ± 0.64 CFU ml^−1^ in the control group, while the phage-treated group showed a significant reduction (*p* = 0.001, CI: 4.64 to 9.97) of bacterial burden to log_10_ 1.18 ± 0.64 CFU ml^−1^ (Fig. 6D). The mean difference between the groups was log_10_ 7.15 ± 0.91 (Fig. 6D). No bacteria were detected in 6 out of the 9 phage-treated samples.

RT-qPCR analysis of cytokine CXCL8 expression (Fig. 6E) showed a 14.17 ± 3.10-fold increase in the infected sample, compared to the not infected control (mean difference = 13.15; 95% CI: -0.05 to 26.34; *p* = 0.051). The treatment with the phage combination strongly reduced the CXCL8 expression (mean difference = 13.02; 95% CI: -0.17 to 26.22; *p* = 0.054). No significant differences were observed among the control, phage-control, and phage-treated groups (all *p* > 0.9). To check the cytokines on protein level a canine ELISA was performed (Fig. 6F). Post hoc analysis revealed a statistically significant increase in CXCL8 cytokine concentration in the infected vector group (7638 ± 818 pg ml^−1^, 95% CI: 5939 to 9337, *p* = 0.001) compared to not infected control group (2780 ± 829 pg ml^−1^). While the phage-treated group significantly reduced the CXCL8 concentration (2646 ± 815 pg ml^−1^, *p* < 0.001, CI: 970 to 4322) compared to infected vector-treated group.

In the qPCR evaluation of the alarmin SAA1, infected group showed an increased mean of the SAA1 level by 10.01 ± 3.22-fold, while in the phage-treated group it was reduced by 7.1 ± 3.22 fold. However, none of the pairwise comparisons between bacteria and control (mean difference = 9.01; 95% CI: -6.83 to 24.83; *p* = 0.499), phage-treated control (mean difference = 9.07; 95% CI: -6.76 to 24.90; *p* = 0.488), or infected phage-treated (mean difference = 9.04; 95% CI: -6.79 to 24.87; *p* = 0.493) reached statistical significance.

## Discussion

This study demonstrates that the phages JG003 and PTLAW1, both alone and in combination significantly reduce the total biovolume and increase the percentage of dead biovolume on an abiotic surface in vitro. The effectiveness of the phage combination treatment was also demonstrated on biotic surfaces in both in vitro and ex vivo skin models. Marked biofilm structure degradation was observed, confirmed by fluorescence and electron microscopy, indicating the phage’s ability to disrupt early-stage biofilms. Additionally, a reduction in bacterial burden and a significant decrease in proinflammatory mediators in phage-treated samples using a canine epidermal equivalent were shown.

The ability to degrade biofilm of the phage alone and in combination was initially tested in a 96-well plate on an abiotic plastic surface, representing a standard, controlled environment. The relative biofilm volume reduction was shown in all phages. Nevertheless, the combination of the phages did not show a significant benefit compared to phage JG003 alone. One possible explanation is that phage–phage competition can reduce the efficacy of co-infections, a phenomenon also observed by Bürkle et al.^26^. Bürkle et al. reported that the phage with the shorter lysis time depletes bacterial resources and induces host mutations, disadvantaging the second phage. This published mechanism likely explains the lack of synergy observed in our study. The significant increase in PI-stained biofilm volume observed in phage-treated groups reflects extensive membrane integrity loss, with a membrane depolarization and collapse of intracellular ATP pool^27^. Furthermore, the volumetric overlap of SYTO 9 and PI signals reflects regions of the biofilm containing membrane-compromised cells, indicating areas where bacterial membranes have been disrupted by phage treatment. While some studies caution that SYTO 9/PI may underestimate viability, the magnitude of our PI signal increase suggests a true biological effect, consistent with recent imaging-based validation. Although the benefits of using the phage combination were not demonstrated in the adherent biofilm, likely due to biofilm-specific protective mechanisms such as reduced phage penetration, metabolic heterogeneity, and dormancy, the results of our previous study^28^in planktonic culture indicated that the combination of these two phages led to a delayed and reduced regrowth compared to single phages. This suggests that the combination may limit the development of bacterial resistance. These observations are also supported by numerous studies^29,30^, highlighting the potential benefit of phage cocktails. The observed biofilm-degrading ability of the phages may be attributed to their genomic potential to encode biofilm-degrading enzymes, as demonstrated in our previous study^28^. Genome analysis revealed that JG003 carries a putative cell wall hydrolase, while PTLAW1 encodes a putative chitinase and lytic transglycosylase, suggesting that these enzymes could contribute to biofilm matrix disruption. Based on this evidence, we chose to focus on testing the phage combination in the more complex skin model experiments, where their anti-biofilm effects could be further evaluated under physiologically relevant conditions. The phages were applied at a high titer (10^9^ PFUmL^−1^), chosen based on the high titer in our previous study^28^and reflecting concentrations commonly used in clinical settings, particularly for topical applications to wound infections, ensuring sufficient activity at the infection site^31,32^.

In the ex vivo model, phage treatment led to a substantial reduction in bacterial burden, though complete eradication was not achieved. Despite a pronounced 4-log reduction in bacterial burden and statistically significant differences between groups, the level of significance was moderate due to biological variability inherent to the ex vivo skin model. The model successfully replicated the complexity of physiological infections by forming a biofilm on canine skin. Intense fluorescence signals observed in the immunofluorescence staining indicate bacterial colocalization, which can be interpreted as biofilm formation and is confirmed by SEM showing matrix-like material connecting the bacteria. SEM additionally reveals a heterogeneous distribution of bacterial aggregates, with areas of dense clusters interspersed with regions of sparse colonization across the tissue surface and a highly complex overall surface structure of the skin which likely explains a few residing bacterial aggregates in the phage-treated group as well despite the fact that CFU counts are clearly reduced. Therefore, SEM provides detailed structural information, however it is not ideal for quantification due to sample preparation artifacts and limited depth resolution^33^.

In our in vitro experiments, we established a canine epidermal equivalent. The phage treatment showed a lower bacterial burden, with the most consistent effect observed for CXCL8, while changes in SAA1 were less pronounced. The absence of detectable bacteria in 6 out of 9 samples may be attributed to the earlier administration of phage treatment, which likely limited bacterial establishment and the formation of biofilm structures. These results indicate that phage therapy has potential as a safe and effective intervention. Notably, phage treatment was applied at an early stage, when bacteria had initiated irreversible surface attachment and early microcolony formation^34,35^, focusing on the inhibition of the development of a mature biofilm.

Our results align with previous studies reporting that phage therapy in infection models ex vivo or cell culture experiments can substantially reduce bacterial populations, but may not achieve complete eradication, particularly in biofilm-associated infections^24,36,37^. This reflects the challenges of achieving complete eradication in biofilm-associated skin infections.

Overall, we were able to mimic a skin infection with biofilm formation. Nevertheless, ex vivo and in vitro experiments have their limitations. In particular, phage efficacy in this study was assessed during early-stage biofilm formation, which does not fully reflect mature biofilms characterized by increased structural complexity and pronounced metabolic heterogeneity. Despite this, phages, especially those encoding depolymerases, have been shown to penetrate and disrupt established biofilms^38,39^, indicating that similar trends of biofilm reduction in mature biofilms, although the magnitude of the effect may differ. Additionally, both systems lack an immune system, that would alter the complex infection process, as well as the outcome of the phage treatment, due to the synergistic effect of the host immune system and the phages^40^. While the canine skin sample contains all the skin barrier components, translation is limited due to the species-specific differences in skin composition, such as hair follicles and gland density. The in vitro model contains viable keratinocytes, allowing us to test more readouts, but it lacks other skin components. Combining both models provides a more comprehensive overview of the infection process. Building on our findings, future studies could explore the permeability of the skin to phages delivered via different galenic formulations, such as encapsulation or nanocarriers^41^. These advanced delivery systems may enhance phage stability, penetration, and therapeutic efficacy. Current literature increasingly supports the use of phage therapy as a complementary approach to antibiotics. Studies have reported additive, synergistic, and occasionally antagonistic interactions between phages and antibiotics in vitro 42^45^, while in vivo experiments have demonstrated improved therapeutic outcomes with optimized combinations^14^.

In conclusion, our study highlights the potential of phage therapy as a promising strategy for disrupting biofilms and reducing bacterial load in skin infections. It is also emphasising the importance of model complexity. The use of high phage titer, consistent with clinical practice, likely contributed to the observed efficacy and supports the translational relevance of our findings. While phage efficacy was primarily evaluated during early-stage biofilm formation, activity against fully mature biofilms remains to be determined, as our current models are designed to capture early biofilm development. In addition, we did not experimentally assess different treatment timings or phage combinations, these factors may be important for achieving complete bacterial eradication and should be explored in future studies. Importantly, established in vitro and ex vivo skin models also provide a valuable platform for investigating the co-evolution of phages and bacteria in the presence of antiseptics and antibiotics. This enables the development of more robust, and resistance-aware therapeutic approaches.

## Materials and methods

### Phages and bacterial isolates

The phages used in this study were previously isolated and characterized^28^and selected based on their lytic activity, and their ability to significantly reduce bacterial regrowth observed when they were applied in combination, their low genetic similarity and their encoding of potentially biofilm-degrading enzymes. These phages were diluted to the appropriate titer using sterile PBS. For the infection experiment, the clinical *Pseudomonas aeruginosa* isolate IMT45060 was used, provided by the Institute of Microbiology and Epizootics of the FU Berlin.

### Biofilm degradation assay

The bacteria were grown in LB medium to an OD of approximately 0.5 (37 °C, 200 rpm) and further diluted to an OD of 0.02. Each well of the 96-well plate (µ-Plate 96 Well Square, ibiTreat, Cat. no. 89626) was filled with 175 µl of bacterial suspension and sealed with the Breathe-Easy^®^ sealing membrane (Cat. No. Z380059, Sigma) and incubated for 16 h (CO_2_ 5%, 37 °C). The wells were treated with 70 µl phage suspension, consisting of JG003 or PTLAW1 (1 × 10^9^ PFU ml^−1^) or the phage combination with a final concentration of 1 × 10^9^ PFU ml^−1^, corresponding to 5 × 10^8^ PFU mL^−1^ per phage. LB medium was used as a control. Thirty-five µl LIVE/DEAD staining solution (BacLight Bacterial Viability Kit, Cat. No. L7012, ThermoFisher Scientific) was added to achieve a final concentration of 5 µM SYTO 9 and 30 µM propidium iodide. They were again sealed with the Breathe-Easy^®^ sealing membrane and incubated for additional 24 h (CO_2_ 5%, 37 °C). The assay was carried out with 2 wells for each group and 3 biological replicates.

### Confocal microscopy, image processing and quantification of biofilm parameter

Established biofilms were analyzed by confocal microscopy using an inverted Stellaris 8 FALCON system (Leica Microsystems) and biofilm parameters were subsequently quantified automatically using BiofilmQ version 1.0.1.^46^. Detailed information about the configuration of the confocal microscope, imaging parameters and image analysis/quantification is described in detail in Text S2 in the supplemental methods file.

### Franz-type diffusion cells setup

The canine full-thickness skin, collected from the Institute of Pathology (FU Berlin), was stored at -80 °C until tested. For the experiment, the skin was thawed, clipped and further processed with multiple tape striping. The upper layer of skin, set to 700 μm, was ablated using an electric dermatome (Acculan 3TI Dermatome, Aesculap) and sectioned to the required diameter (approx. 12 mm) with a scalpel. The skin sections were disinfected in 2.5% Povidone-iodine solution for one minute, rinsed thrice, and hydrated in PBS for 15 min. The sections were clamped in the sterile Franz-type diffusion cell (Gauer Glas Püttlingen, Germany) between the donor and the acceptor chamber, with the epithelium facing the donor compartment. The acceptor chamber was filled with PBS (+ 0.1% BSA). The Franz-type diffusion cells were placed on a magnetic stirrer and connected to a water bath. The resulting temperature consistency on the skin surface was monitored using an infrared thermometer. All experiments were performed with 2 technical replicates and 3 biological replicates.

### Infection experiment in Franz-type diffusion cells

The skin was infected with 150 µl of a *P. aeruginosa* culture (IMT45060) with an OD_600_ of 0.5 (4 × 10^7^ CFU in 150 µl LB-media), sterile LB-media as sterility control, sealed with Parafilm® to avoid evaporation and incubated for 16 h. After testing the thermal constancy using infrared thermometer, the skin was treated with 100 µl of a phage combination with a total titer of 10^9^ PFU ml^−1^ (corresponding to 5 × 10^8^PFU mL^−1^ per phage ) or sterile PBS. After additional 8 h of incubation, the temperature was measured and the samples taken with a biopsy punch (6 mm) and transferred into 4% formaldehyde (ROTI® Histofix) for histological examination, in 1 ml PBS for bacterial and phage quantification and in electron microscopy buffer for the SEM analysis. Samples from the acceptor chamber were taken for phage and bacteria detection.

### Enumeration of phages and bacteria of the Franz-type diffusion cells

The weighted skin samples in 1 ml PBS were homogenized on ice with a dispersing instrument (ULTRA-TURRAX® T 10 basic), placed in an ultrasonic bath for 10 min and vortexed for 1 min. Serial dilutions were performed before plating on selective CFC-Agar (Oxoid) plates and incubation overnight at 37 °C. For enumeration of the phages, the skin samples were pelleted for 10 min at 10,000  ×*g* (4 °C), the supernatant was filtered (0.22 μm, Supor membrane, PALL) and examined in a spot assay, as previously described^28^. The samples from the acceptor chamber were plated on selective CFC agar plates and by spot assay.

### Histology

The skin biopsies were fixed in 4% formaldehyde (ROTI® Histofix) for at least 24 h. For processing, the samples were dehydrated and embedded in paraffin, and 4-µm slices were prepared. Haematoxylin and eosin (H&E) staining was performed according to the standard protocol. The thickness of the sections was measured at ten positions per section using a light microscope (×40 objective Nikon Plan Fluor, Nikon Eclipse Ni-E), and digital images were captured using NIS Elements AR software (5.30.02).

### Immunofluorescence staining

Immunostaining of 4 μm paraffined skin sections was performed using antibodies against pan-cytokeratin and *P. aeruginosa*, followed by fluorescence microscopy, as detailed in Text S3 in the supplemental methods file.

### Canine epidermal equivalent

For the preparation of the canine epidermal equivalent (EE), the cell line canine epidermal keratinocyte progenitors (CPEK) were seeded (300 000 cells/transwell) in the upper compartment of a transwell insert system (6.5 mm transwell, 0.4 μm pore polyester membraneCorning) in 100 µl canine epithelial proliferation medium (CnT-09, CellnTec). After 24 h the cells were lifted at an air-liquid-interface (ALI). The cells were cultured with ALI for additional 14 days with media change every 2 days in an incubator (37 °C, 5% CO_2_). All experiments were performed in 3 technical replicates and 3 biological replicates.

### Canine epidermal equivalent infection experiment

The canine EE was infected with a concentration of 1 × 10^3^ CFU in 30 µl LB-media of the clinical isolate *P. aeruginosa* (IMT45060) and incubated at 37 °C and 5% CO_2_. After 3 hours, the transwells were treated with 30 µl of phage combination (JG003 and PTLAW1) with a total of 10^9^ PFU ml^−1^, corresponding to 5 × 10^8^PFU mL^−1^ per phage, or sterile PBS. The samples from the EE were taken after a total incubation time of 16 h. The samples for histological examination were transferred into 4% formaldehyde (ROTI^®^ Histofix), in 200 µl 1% Triton X for bacterial enumeration. The samples for the SEM were fixed using glutaraldehyde vapor fixation with 25% glutaraldehyde for 2 × 1 h, followed by exposure to Karnovsky vapor for transport. For the quantitative qPCR, the EE were lysed in Buffer RLT (Qiagen) with 1% β-mercaptoethanol and stored at -80 °C. Samples from the lower compartment were taken for phage and bacteria detection and ELISA measurements.

### Scanning electron microscopy

The SEM sample preparation was performed as previously described^47^. Additional information is available in the supplemental methods file in Text S4.

### Enumeration of phages and bacteria of the epidermal equivalent

The EE were resuspended in 1% Triton X-100 and placed in an ultrasonic bath for 10 min, followed by 1-minute vortexing. For bacteria enumeration a serial dilution was performed before plating on LB plates and incubation overnight at 37 °C. The phages were enumerated as previously described for the skin biopsies. The samples from the acceptor chamber were plated on LB plates for bacterial detection and on spot assays for phage detection.

### RNA extraction and RT-qPCR

The RNA isolation was performed with RNeasy Mini Kit (Qiagen, Hilden, Germany), followed by DNA transcription and qPCR using the Maxima SYBR Green/Fluorescein qPCR Master Mix (Thermo Fisher Scientific Baltics UAB, Vilnius, Lithuania). The genes RPL13A and GAPDH were used as reference genes for normalization. Details about the protocol can be found in supplementary methods file (Text S5).

### ELISA

The Cytokine CXCL8 concentration in the media of the lower compartment in the EE experiment was measured according to the manufactures protocol (Quantikine, DuoSet ELISA Development Systems, R&D Systems) in duplicates. The appropriate dilutions were determined in preliminary tests (data not shown).

### Data presentation and statistical analysis

For the statistical analysis and graphical representation, GraphPad Prism 10.0.2 was used unless otherwise specified. The statistical analysis of biofilm degradation was performed using ordinary one-way ANOVA, followed by Dunnett’s multiple comparisons test. The CFU ml^−1^ were normalized as log values due to the non-normal distribution and statistically examined using the unpaired t-test. The CXCL8 ELISA results were screened for outlier’s trough ROUT method (Q = 1%). One value (bacteria + phages: 17783.82 pg ml^−1^) was identified as outlier and subsequently excluded from the statistical analysis. The data of the PCR and ELISA were analyzed using a linear mixed-effects model in SPSS (version 29.0.0.0), with groups as fixed factor and transwell as a random factor. The post hoc comparisons between treatment groups were performed using Bonferroni. A significance level of α = 0.05 was used. The graphs are shown as Mean ± SD. Figures were partially created using BioRender.com.

## Supplementary Information

Below is the link to the electronic supplementary material.

Supplementary Material 1

Supplementary Material 2

Supplementary Material 3

Supplementary Material 4

## Data Availability

All data supporting the findings of this study are available within the article and its supplementary materials.

## Abbreviations

PBS: Phosphate Buffered Saline
SEM: Scanning electron microscopy
CLSM: Confocal Laser Scanning Microscopy
CXCL8: CXC-Motiv-Chemokin 8
SAA1: Serum amyloid A1
EE: Epidermal equivalent
