# Supplementary material for: Evaluation of bacteriophage efficacy against Pseudomonas aeruginosa in ex vivo and in vitro canine skin systems
Source: Sci Rep. 2026 Feb 17;16:7167. doi: 10.1038/s41598-026-40091-8 (PMC12920615; doi:10.1038/s41598-026-40091-8)
Supplement: Supplementary file 1 — Supplementary Material 1 [file 41598_2026_40091_MOESM1_ESM.pdf]

Supplementary figure 2:

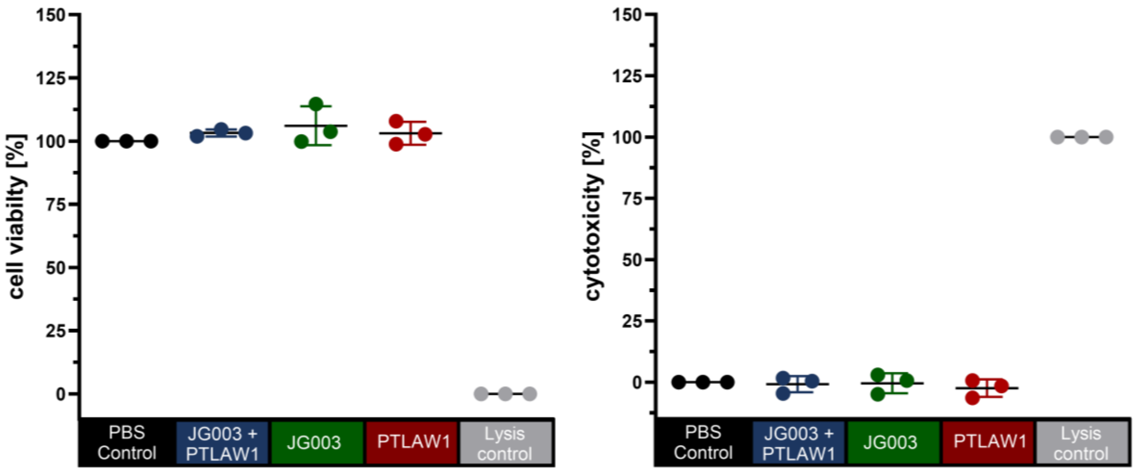

Cytotoxicity and viability of bacteriophage treatments in CPEK cells. Canine keratinocytes (CPEK) were cultured for 24 hours and subsequently treated with bacteriophages JG003, PTLAW1, or their combination for an additional 24 hours. Cell viability and cytotoxicity were assessed using the CellTiter 96® Cell Proliferation Assay and CellTox™ Green Cytotoxicity Assay, respectively. Results are presented as percentage viability and percentage cytotoxicity (Mean ± SD). All experiments were performed in biological triplicates.
