# Supplementary material for: Evaluation of bacteriophage efficacy against Pseudomonas aeruginosa in ex vivo and in vitro canine skin systems
Source: Sci Rep. 2026 Feb 17;16:7167. doi: 10.1038/s41598-026-40091-8 (PMC12920615; doi:10.1038/s41598-026-40091-8)
Supplement: Supplementary file 2 — Supplementary Material 2 [file 41598_2026_40091_MOESM2_ESM.pdf]

Supplementary figure 3:

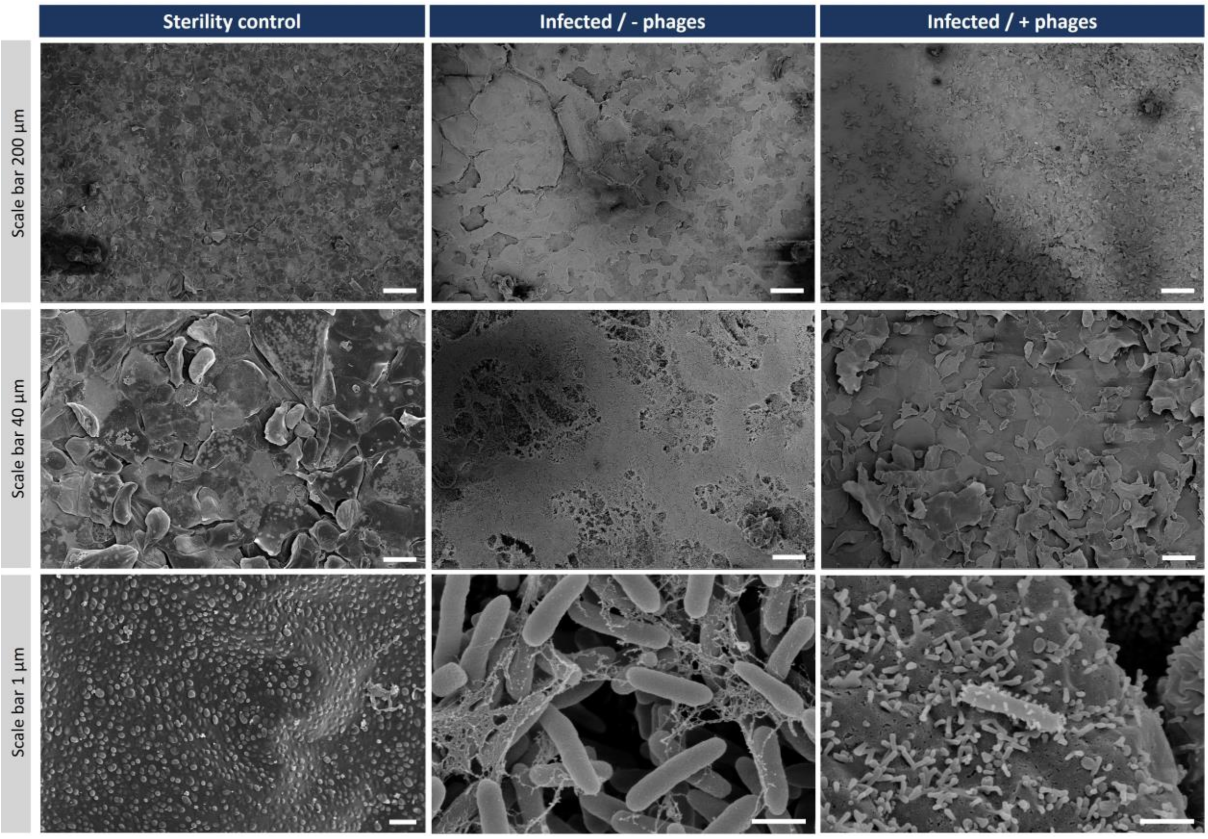

SEM micrographs of phage treatment of *P. aeruginosa* in the canine epidermal equivalent (EE) model. Canine keratinocytes were cultured in a transwell ALI system for 14 days and infected with *P. aeruginosa* (1×10^3 CFU). After 3 hours, the phage cocktail (JG003 + PTLAW1, 1×10^9 PFUmL^{-1}) was applied and incubated for an additional 13 hours. For improved visualization, contrast and brightness of the micrographs was adjusted.
