# Supplementary material for: Evaluation of bacteriophage efficacy against Pseudomonas aeruginosa in ex vivo and in vitro canine skin systems
Source: Sci Rep. 2026 Feb 17;16:7167. doi: 10.1038/s41598-026-40091-8 (PMC12920615; doi:10.1038/s41598-026-40091-8)
Supplement: Supplementary file 3 — Supplementary Material 3 [file 41598_2026_40091_MOESM3_ESM.pdf]

Supplementary figure 1:

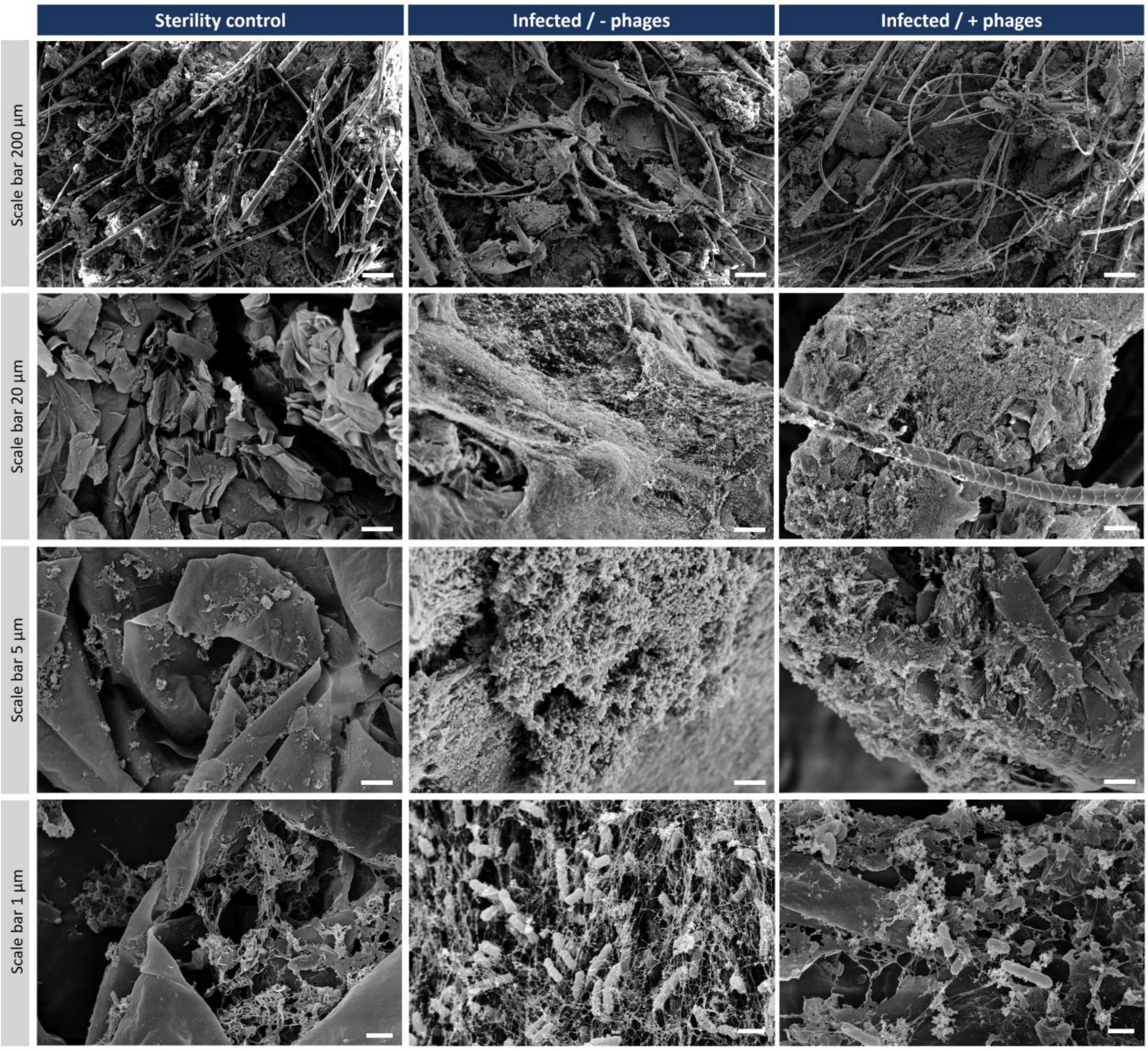

SEM micrographs of phage treatment in the *ex vivo* canine skin model using Franz-type diffusion cells. The skin samples were clamped into the Franz-type-diffusion cells and infected with *P. aeruginosa* for 16 hours, followed by treatment with the phage combination JG003 + PTLAW1 for 8 hours. For improved visualization, contrast and brightness of the micrographs was adjusted. SEM micrographs with a 5 µm scale bar, corresponding to Fig. 3D, are shown here for reference.
