# Supplementary material for: Evaluation of bacteriophage efficacy against Pseudomonas aeruginosa in ex vivo and in vitro canine skin systems
Source: Sci Rep. 2026 Feb 17;16:7167. doi: 10.1038/s41598-026-40091-8 (PMC12920615; doi:10.1038/s41598-026-40091-8)
Supplement: Supplementary file 4 — Supplementary Material 4 [file 41598_2026_40091_MOESM4_ESM.pdf]

Supplementary methods file

Text S1: Cytotoxicity and viability of bacteriophage treatments in CPEK cells

The cytotoxic potential of individual bacteriophages (JG003 and PTLAW1) and their combination was evaluated on canine progenitor epidermal keratinocytes (CPEK) using the CellTox™ Green Cytotoxicity Assay (Promega). Cell viability was assessed in parallel using the MTT assay (CellTiter 96®, Promega). CPEK cells were seeded at densities of 2,500 or 5,000 cells per well in 96-well plates (Well Black Plate, Thermo Scientific) and incubated for 24 hours (37°C, 5% CO_2). Following incubation, the culture medium was replaced with CellTox™ Green in fresh medium (CnT-09, CellnTec) according to the manufacturer's instructions. Cells were then treated with bacteriophages at a final concentration of 1×10^9 PFU/mL and incubated for an additional 24 h (37°C, 5% CO_2). Maximum cytotoxicity (lysis control) was induced using the lysis solution provided with the CellTox™ Green assay kit, according to the manufacturer's protocol. Fluorescence was measured at an excitation wavelength of 485 nm and an emission wavelength of 525 nm using a microplate reader (ALF, Thermo Scientific). Following, CellTiter 96® reagent was added to each well and the plate was incubated for an additional 2 hours (37°C, 5% CO_2). The substance was transferred to a clear plate and the absorbance was measured at 490 nm using a microplate reader (ALF, Thermo Scientific). Cytotoxicity and cell viability in % were calculated as percentages relative to the PBS control (negative control) and the lysis control, respectively. All experiments were performed in biological triplicate.

Text S2: Confocal Microscopy, image processing and quantification of biofilm parameter

Confocal microscopy was done with an inverted Stellaris 8 FALCON system (Leica Microsystems), equipped with a 405 nm laser, a tunable white light laser (WLL, 440 – 790 nm), and Power HyD detectors. The microscope was operated by LAS X software version 4.8.1.29271. Images were acquired with a HC PL APO CS2 40×/1.10 water immersion objective. The following settings for fluorescence signal detection were used: Syto 9 was excited at 488 nm with an intensity of 0.38% and emission was detected between 505 – 560 nm with detector gain of 2.5 in intensity mode; PI was excited at 561 nm with an intensity of 1.5% and emission was detected between 575 – 655 nm with a detector gain of 2.5 in intensity mode. Line sequential acquisition mode without averaging at 600 Hz scan speed and the pinhole set to 1 airy unit was used for acquisition. Z-stacks of a total height of 51 µm with a step size of 3 µm were acquired in 3 positions in the middle of each the well. Starting points of the stacks were automatically found by a reflection based autofocus step on the bottom of the well with an offset of -5 µm. Stacks were acquired with a zoom of 1 and a pixel size of 0.284 µm, resulting in a physical size of 291 × 291 µm.

For display purposes, 3D image stacks were rendered in LAS X 3D version 4.8.1. (Leica Microsystems) in `Maximum´ mode. Values for minimum and maximum were set identical for all images. Automatic quantification of biofilm parameter was done with BiofilmQ version 1.0.1. Image stacks were preprocessed by applying a mean filter with a kernel size of 11 pixel (xy) and 3 pixel (z) and segmentation artefacts introduced by floating cells were suppressed by using the floating cell suppression algorithm of BiofilmQ [1]. Subsequently, segmentation was performed with `RobustBackground´ algorithm using a sensitivity of 0.35 for the SYTO 9 channel and a sensitivity of 0.7 for the PI channel, respectively. The biofilms were further segmented into cubes with a side length of 15 pixel. Post-processing of the masks consisted of a 3D median filter and the removal of objects with a size smaller than 1000 voxels. Subsequently, the biofilm volume of the SYTO 9 and PI channel was calculated. In addition, the total biofilm volume after merging the segmentation masks of both channels as well as the normalized overlap between SYTO 9 and PI in the total biofilm volume was determined.

Text S3: Immunofluorescence staining

The skin samples (4 µm sections) were immunostained using antibodies for pan-cytokeratin and *P. aeruginosa*. For the staining, sections were deparaffinized, placed in citrate buffer (pH 6) at 95 °C for 20 min, cooled at room temperature and rinsed with PBS. After permeabilization step (PBS, 0.2 % Triton X-100) the slides were blocked to prevent unspecific binding (Roti-Immunoblock, 20% donkey serum) for 30 minutes. Sections were incubated with anti-pan-cytokeratin antibodies (AE1/AE3, sc-81714, 1:50) and anti-pseudomonas antibody (PA1-73116, 1:800) in PBS with 2% BSA overnight at 4°C, followed by the incubation of secondary antibody (Alexa-Fluor® anti-rabbit 647 and Alexa-Fluor® anti-mouse 488, 1:800) for 30 min. After washing steps with PBS, the stains were mounted with Fluoromount G with DAPI (Invitrogen). Buffer control and isotype control Immunoglobulin G (DAKO, X0936, dilution 1:800) were used to set the exposure time. For visualization the microscope Leica DMi8 with the LAS X Software (3.5.7) was used with the objective HC PL FLUORTARL 20x/0.40 Dry for the canine skin samples and HC PL FLUOTAR L 40x/0.60 dry for the epidermal equivalent. Image processing was performed using Fiji software (version 1.54p) [2].Brightness and contrast were adjusted using blank/control samples as a reference to ensure consistent visualization across all images.

Text S4: Scanning electron microscopy

Samples of the Franz-type diffusion cell were fixed in 5% formaldehyde and 2% glutaraldehyde in 0.1 M HEPES buffer (HEPES 0.1 M, 0.09 M sucrose, 10 mM $CaCl_2$, 10 mM $MgCl_2$, pH 6.9) at 4° C or with glutaraldehyde vapour (see canine EE equivalent infection model description). Next, samples were

washed twice with TE-buffer, pH 7.0 (20 mM Tris/HCl, 1.0 mM EDTA) and dehydrated in a graded series of ethanol for 15 min at each step. Afterwards, samples were critical-point dried (CPD 300, Leica Microsystems, Wetzlar) and sputter coated with a gold-palladium film (SCD 500, Bal-Tec, Lichtenstein) before examination in a field-emission scanning-electron microscope Merlin (Zeiss, Oberkochen, Germany) using the Everhart Thornley SE detector and the SE inlens detector at different ratios and with an acceleration voltage of 5 kV.

Text S5: RNA extraction and RT-qPCR

The mRNA isolation was performed with the RNeasy Mini Kit (Qiagen, Hilden, Germany) with additional DNA digestion with DNase-Free DNase Kit (Qiagen), according to manufacturer's protocol. The QuantiTect Reverse Transcription Kit (Qiagen) was used for transcription of the RNA in cDNA. For the qPCR the Maxima SYBR Green/Fluorescein qPCR Master Mix (2X) was mixed with 200 ng of cDNA (Thermo Fisher Scientific Baltics UAB, Vilnius, Lithuania) and 1 µl of 1 µM reverse and forward primer and run with the following protocol: After an initial denaturation period at 95°C for 10 minutes, 40 cycles were performed. The cycles included denaturation at 9 5°C for 15 s, annealing for 30 s at 60°C and extension at 72°C for 30 s. Melting curves were evaluated for quality investigation and gels were run to prove the right amplification size. As target genes the chemokines CXCL6 and CXCL8 as well as serum amyloid A1 (SAA1) were tested (Supplementary Table 1). The genes RPL13A and GAPDH were used as reference genes for normalization factor. The calculation of the $2{-}\Delta Cq$ method was performed according to [3]. The primer efficacy was tested before carried out the experiment (>95 %).

Supplementary table 1: Primer Sequences

Gene	Forward sequence	Reverse sequence	NCBI database
GAPDH	GGGAAACTTGTCATCAACGGG	GCCTTTCAAGTGAGCCCCAG	NM_001003142.2
RPL13A	AGAAGAAACAGCTTATGAGGCTAC	CCAGGCCAAGCATGATGAATAA	NM_001313766.4
SAA1	GCTGCTAAAGTGATCAGCGAC	GAAGTGGTTGGGGTCTTTGC	XM_038430647.1
CXCL8	TGGCAGCTTTTGTCCTTTCT	GGGCCACTGTCAATCACTCT	ENSCAFG00000003029.5

References

1. Hartmann R, Jeckel H, Jelli E, Singh PK, Vaidya S, Bayer M, et al. Quantitative image analysis of microbial communities with BiofilmQ. Nat Microbiol. 2021;6(2):151-6; doi: 10.1038/s41564-020-00817-4.
2. Schindelin J, Arganda-Carreras I, Frise E, Kaynig V, Longair M, Pietzsch T, et al. Fiji: an open-source platform for biological-image analysis. Nat Methods. 2012;9(7):676-82; doi: 10.1038/nmeth.2019.
3. Schmittgen TD, Livak KJ. Analyzing real-time PCR data by the comparative CT method. Nature Protocols. 2008;3(6):1101-8; doi: 10.1038/nprot.2008.73.
